# Supplementary material for: Gender, Age, Family and Territorial Features of Dietary and Physical Activity Patterns in Russian Youths
Source: Int J Environ Res Public Health. 2022 May 9;19(9):5779. doi: 10.3390/ijerph19095779 (PMC9104441; doi:10.3390/ijerph19095779)
Supplement: Supplementary file 1 [file ijerph-19-05779-s001.zip › ijerph-1686703-supplementary/Supplementary Table S1.pdf]

Supplementary Table S1. Definitions of variables used in the study.

Variable	Definitions and Grouping
**Independent variables used in the analysis**	
Gender	Boy; girl
Age	10–12, 13–15, 16–17 years old
Family composition	Incomplete family – only 1 parent; complete family – two parents
Number of children in the family	1 child; 2 or more children
Mother’s education	Education other than higher; higher education
The presence of smoking adults in the family	There are no smokers in the family; there are smokers in the family
Availability of a private room	The child participating in the study lives in a separate room from other family members; the child lives in a shared room with another member of the family
**Variables used for forming dietary patterns and physical activity patterns**	
Frequency of consumption of fruits and vegetables	4 grades: “Once a day or several times a day” – 1 point, “3–4 times a week” – 2 points, “Once a week or less” – 3 points, “I do not consume” – 4 points
Frequency of fast food consumption	
Frequency of consumption of hot meals (excluding fast food)	
Frequency of consumption of carbonated drinks	
Frequency of consumption of meat products (including poultry)	
Frequency of consumption of fish and fish products	
Frequency of consumption of milk and dairy products	
The frequency of consumption of smoked meats	2 grades: “None/Not sure” – 1 point, “I consume” – 2 points.
Frequency of consumption of canned foods	
Daily number of meals	2 grades: «1–2 times a day or every day is different” – 2 points and “3–5 times a day” – 1 point.
Availability of breakfast	5 grades: “Every day” – 1 point, “School days only” – 2 points, “Weekends only” – 3 points, “Rarely” – 4 points, “Never” – 5 points.
Sleep duration	Quantified in hours and minutes, with subsequent conversion to minutes per day
The question, “Do you do morning exercises?”	3 grades: “No” – 3 points, “Once in a while” – 2 points, “Yes, always” – 1 point
Attending school physical education lessons	2 grades: “No” – 2 points, “Yes” – 1 point
The question, “How often do you walk outdoors?”	3 grades: “Every day” – 1 point, “Weekends only” – 2 points, “I mostly stay at home” – 3 points.
