# Supplementary material for: Gender, Age, Family and Territorial Features of Dietary and Physical Activity Patterns in Russian Youths
Source: Int J Environ Res Public Health. 2022 May 9;19(9):5779. doi: 10.3390/ijerph19095779 (PMC9104441; doi:10.3390/ijerph19095779)
Supplement: Supplementary file 1 [file ijerph-19-05779-s001.zip › ijerph-1686703-supplementary/Supplementary Table S2.pdf]

Supplementary Table S2.1. Factor loadings of main identified dietary and PA patterns (boys).

Parameters	Identified Patterns			
	**DP 1**	**DP 2**	**DP 3**	**PAP**
Fruits and vegetables	0.41	-	0.50	-
Hot meals	0.85	-	-	-
Meat and meat products	0.65	-	-	-
Fish and fish products	0.51	-	-	-
Milk and dairy products	0.70	-	-	-
Fast food	-	−0.82	-	-
Carbonated drinks	-	−0.82	-	-
Smoked meats and canned food	−0.46	0.40	-	-
Meal frequency	-	-	0.72	-
Availability of breakfast	-	-	0.73	-
Sleep duration	-	-	-	−0.88
Morning exercises	-	-	-	0.49
Physical education lessons	-	-	-	0.58
Outdoor walks	-	-	-	0.78
Explained variance, %	0.24	0.15	0.15	0.30

Note: DP – dietary pattern, PAP – physical activity pattern.

Supplementary Table S2.2. Factor loadings of main identified dietary and PA patterns (girls).

Parameters	Identified Patterns			
	**DP 1**	**DP 2**	**DP 3**	**PAP**
Fruits and vegetables	0.49	-	-	-
Hot meals	0.56	-	-	-
Meat and meat products	0.65	-	-	-
Fish and fish products	0.56	-	-	-
Milk and dairy products	0.44	-	-	-
Fast food	-	−0.72	-	-
Carbonated drinks	-	−0.76	-	-
Smoked meats and canned food	-	0.54	-	-
Meal frequency	-	-	0.80	-
Availability of breakfast	-	-	0.78	-
Sleep duration	-	-	-	−0.75
Morning exercises	-	-	-	0.40
Physical education lessons	-	-	-	0.55
Outdoor walks	-	-	-	0.28
Explained variance, %	0.16	0.16	0.13	0.28

Note: DP – dietary pattern, PAP – physical activity pattern.

Supplementary Table S2.3. Factor loadings of main identified dietary and PA patterns (Moscow).

Parameters	Identified Patterns			
	DP 1	DP 2	DP 3	PAP
Fruits and vegetables	0.61	-	-	-
Hot meals	0.39	-	-	-
Meat and meat products	0.48	-	-	-
Fish and fish products	0.55	-	-	-
Milk and dairy products	0.60	-	-	-
Fast food	-	−0.81	-	-
Carbonated drinks	-	−0.78	-	-
Smoked meats and canned food	-	0.55	-	-
Meal frequency	-	-	0.81	-
Availability of breakfast	-	-	0.81	-
Sleep duration	-	-	-	−0.78
Morning exercises	-	-	-	0.46
Physical education lessons	-	-	-	0.60
Outdoor walks	-	-	-	-
Explained variance, %	0.15	0.17	0.14	0.30

Note: DP – dietary pattern, PAP – physical activity pattern.

Supplementary Table S2.4. Factor loadings of main identified dietary and PA patterns (Murmansk).

Parameters	Identified Patterns			
	DP 1	DP 2	DP 3	PAP
Fruits and vegetables	-	-	0.58	-
Hot meals	0.53	-	-	-
Meat and meat products	0.76	-	-	-
Fish and fish products	0.29	-	0.37	-
Milk and dairy products	0.47	-	-	-
Fast food	-	−0.80	-	-
Carbonated drinks	-	−0.81	-	-
Smoked meats and canned food	−0.53	-	-	-
Meal frequency	-	-	0.70	-
Availability of breakfast	-	-	0.73	-
Sleep duration	-	-	-	−0.83
Morning exercises	-	-	-	0.79
Physical education lessons	-	-	-	0.37
Outdoor walks	-	-	-	0.62
Explained variance, %	0.15	0.15	0.16	0.29

Note: DP – dietary pattern, PAP – physical activity pattern.

Supplementary Table S2.5. Factor loadings of main identified dietary and PA patterns (10–12 y/o).

**Parameters**	**Identified Patterns**			
	**DP 1**	**DP 2**	**DP 3**	**PAP**
Fruits and vegetables	0.63	-	-	-
Hot meals	0.51	-	-	-
Meat and meat products	0.69	-	-	-
Fish and fish products	0.74	-	-	-
Milk and dairy products	0.77	-	-	-
Fast food	-	−0.78	-	-
Carbonated drinks	-	−0.77	-	-
Smoked meats and canned food	-	0.63	-	-
Meal frequency	-	-	0.78	-
Availability of breakfast	-	-	0.75	-
Sleep duration	-	-	-	−0.67
Morning exercises	-	-	-	0.81
Physical education lessons	-	-	-	0.65
Outdoor walks	-	-	-	0.72
Explained variance, %	0.20	0.18	0.13	0.32

Note: DP – dietary pattern, PAP – physical activity pattern.

Supplementary Table S2.6. Factor loadings of main identified dietary and PA patterns (13–15 y/o).

**Parameters**	**Identified Patterns**			
	**DP 1**	**DP 2**	**DP 3**	**PAP**
Fruits and vegetables	0.48	-	-	-
Hot meals	-	-	0.64	-
Meat and meat products	0.56	-	-	-
Fish and fish products	0.44	0.45	-	-
Milk and dairy products	0.68	-	-	-
Fast food	-	−0.79	-	-
Carbonated drinks	-	−0.80	-	-
Smoked meats and canned food	−0.44	-	-	-
Meal frequency	-	-	0.60	-
Availability of breakfast	-	-	0.74	-
Sleep duration	-	-	-	−0.53
Morning exercises	-	-	-	0.70

Physical education lessons	-	-	-	0.48
Outdoor walks	-	-	-	0.54
Explained variance, %	0.14	0.17	0.15	0.29

Note: DP – dietary pattern, PAP – physical activity pattern.

Supplementary Table S2.7. Factor loadings of main identified dietary and PA patterns (16–17 y/o).

**Parameters**	**Identified Patterns**			
	**DP 1**	**DP 2**	**DP 3**	**PAP**
Fruits and vegetables	0.43	-	-	-
Hot meals	0.65	-	-	-
Meat and meat products	0.54	-	0.39	-
Fish and fish products	0.64	-	-	-
Milk and dairy products	0.54	-	-	-
Fast food	-	−0.80	-	-
Carbonated drinks	-	−0.82	-	-
Smoked meats and canned food	-	-	-	-
Meal frequency	-	-	0.82	-
Availability of breakfast	-	-	0.81	-
Sleep duration	-	-	-	−0.47
Morning exercises	-	-	-	0.66
Physical education lessons	-	-	-	0.95
Outdoor walks	-	-	-	0.77
Explained variance, %	0.18	0.15	0.15	0.34

Note: DP – dietary pattern, PAP – physical activity pattern.
