# Supplementary material for: Gender, Age, Family and Territorial Features of Dietary and Physical Activity Patterns in Russian Youths
Source: Int J Environ Res Public Health. 2022 May 9;19(9):5779. doi: 10.3390/ijerph19095779 (PMC9104441; doi:10.3390/ijerph19095779)
Supplement: Supplementary file 1 [file ijerph-19-05779-s001.zip › ijerph-1686703-supplementary/Supplementary Table S3.pdf]

Supplementary Table S3. Associations in strata by sex, age, place of residence.

10–12 YEARS OLD		Third tertile DP1		Third tertile DP2		Third tertile DP3		Third tertile PAP	
		**OR**	**95% CI**	**OR**	**95% CI**	**OR**	**95% CI**	**OR**	**95% CI**
Sex, (ref. women)	Man	1.27	0.68–2.37	0.48 *	0.23–0.99	1.17	0.62–2.24	0.89	0.36–2.18
City, (ref. Moscow)	Murmansk	0.94	0.52–1.70	3.64 *	1.84–7.20	1.81	0.99–3.30	1.70	0.74–3.90
Living conditions, (ref. Separate room)	Shared room	1.27	0.66–2.44	0.76	0.41–1.39	0.94	0.47–1.87	0.83	0.30–2.30
Family, (ref. Only one parent)	Complete	0.98	0.46–2.08	0.49	0.23–1.05	0.47 *	0.23–0.98	0.97	0.33–2.90
Number of children, (ref. One child)	Two or more	1.43	0.77–2.64	1.04	0.55–1.99	1.25	0.66–2.36	1.01	0.41–2.47
Mother's education (ref. Not higher)	Higher	0.75	0.39–1.43	0.49 *	0.24–0.98	1.06	0.54–2.01	0.72	0.29–1.78
Family smoking (ref. none)	Yes	1.38	0.73–2.62	2.57 *	1.33–4.97	0.81	0.41–1.58	1.03	0.41–2.60
**13–15 YEARS OLD**		**Third tertile DP1**		**Third tertile DP2**		**Third tertile DP3**		**Third tertile PAP**	
		**OR**	**95% CI**	**OR**	**95% CI**	**OR**	**95% CI**	**OR**	**95% CI**
Sex, (ref. women)	Man	1.14	0.70–1.88	2.62 *	1.57–4.35	0.45	0.28–0.72	0.91	0.57–1.44
City, (ref. Moscow)	Murmansk	1.01	0.61–1.66	0.99	0.60–1.64	1.26	0.77–2.04	0.54 *	0.34–0.87
Living conditions, (ref. separate room)	Shared room	1.06	0.56–1.97	1.44	0.78–2.65	1.14	0.63–2.05	0.97	0.54–1.73
Family, (ref. Only one parent)	Complete	1.37	0.73–2.68	0.86	0.46–1.62	0.67	0.37–1.21	0.57	0.32–1.03
Number of children, (ref. One child)	Two or more	0.77	0.47–1.28	0.85	0.52–1.40	1.41	0.87–2.29	0.88	0.55–1.41
Mother's education (ref. Not higher)	Higher	0.82	0.47–1.43	1.02	0.58–1.81	0.76	0.45–1.30	1.21	0.70–2.08
Family smoking (ref. none)	Yes	1.61	0.98–2.64	1.71 *	1.04–2.82	1.74 *	1.07–2.81	0.78	0.48–1.25

16–17 YEARS OLD		Third tertile DP1		Third tertile DP2		Third tertile DP3		Third tertile PAP	
		OR	95% CI	OR	95% CI	OR	95% CI	OR	95% CI
Sex, (ref. women)	Man	0.74	0.41–1.33	3.16 *	1.65–6.09	0.64	0.35–1.18	0.68	0.39–1.21
City, (ref. Moscow)	Murmansk	0.66	0.36–1.22	1.30	0.69–2.45	0.66	0.35–1.25	0.69	0.39–1.24
Living conditions, (ref. separate room)	Shared room	1.75	0.91–3.37	1.16	0.57–2.39	1.69	0.86–3.32	0.79	0.41–1.51
Family, (ref. Only one parent)	Complete	0.78	0.40–1.55	0.73	0.36–1.50	0.92	0.45–1.86	0.81	0.42–1.58
Number of children, (ref. One child)	Two or more	0.86	0.48–1.54	0.49 *	0.26–0.92	1.24	0.68–2.28	1.24	0.70–2.20
Mother's education (ref. Not higher)	Higher	1.01	0.50–2.04	0.49 *	0.24–0.99	0.54	0.27–1.09	0.88	0.45–1.73
Family smoking (ref. none)	Yes	1.48	0.82–2.68	1.00	0.53–1.86	1.05	0.57–1.93	0.55 *	0.31–0.97

FEMALE		Third tertile DP1		Third tertile DP2		Third tertile DP3		Third tertile PAP	
		OR	95% CI	OR	95% CI	OR	95% CI	OR	95% CI
Возраст (реф. 10–12 лет)	13–15 лет	1.16	0.66–2.04	1.31	0.71–2.44	2.74 *	1.57–4.77	5.78 *	2.96–11.24
	16–17 лет	2.14 *	1.16–3.94	1.34	0.67–2.69	1.79	0.96–3.33	8.20 *	4.00–16.80
City, (ref. Moscow)	Murmansk	1.42	0.90–2.25	1.41	0.84–2.36	1.02	0.65–1.60	0.59 *	0.36–0.96
Living conditions, (ref. separate room)	Shared room	1.33	0.81–2.17	1.75 *	1.01–3.05	1.34	0.82–2.18	1.13	0.69–1.88
Family, (ref. Only one parent)	Complete	0.85	0.48–1.49	0.82	0.43–1.52	0.73	0.42–1.26	0.67	0.38–1.19
Number of children, (ref. One child)	Two or more	1.13	0.71–1.79	0.54 *	0.32–0.92	1.39	0.89–2.18	1.10	0.69–1.75
Mother's education (ref. Not higher)	Higher	0.71	0.43–1.17	0.39 *	0.23–0.67	0.57 *	0.35–0.94	0.93	0.55–1.57
Family smoking (ref. none)	Yes	1.24	0.77–2.01	1.97 *	1.13–3.41	1.40	0.88–2.24	0.65	0.39–1.07

MALE		Third tertile DP1		Third tertile DP2		Third tertile DP3		Third tertile PAP	
		OR	95% CI	OR	95% CI	OR	95% CI	OR	95% CI
Возраст (реф. 10–12 лет)	13–15 лет	1.03	0.57–1.86	0.89	0.54–1.48	1.07	0.62–1.84	5.48 *	2.72–11.02
	16–17 лет	1.07	0.62–1.83	0.93	0.53–1.62	0.87	0.47–1.60	6.47 *	3.10–13.51
City, (ref. Moscow)	Murmansk	0.55 *	0.35–0.87	0.80	0.52–1.21	1.31	0.84–2.06	0.80	0.50–1.27
Living conditions, (ref. separate room)	Shared room	1.39	0.81–2.39	0.63	0.37–1.08	1.06	0.60–1.85	0.66	0.35–1.25
Family, (ref. Only one parent)	Complete	1.24	0.71–2.16	064	0.38–1.06	0.60	0.35–1.01	0.68	0.39–1.19
Number of children, (ref. One child)	Two or more	0.77	0.49–1.21	0.99	0.65–1.51	1.12	0.71–1.77	0.98	0.61–1.56
Mother's education (ref. Not higher)	Higher	1.04	0.60–1.81	1.04	0.63–1.73	1.06	0.62–1.84	1.08	0.62–1.90
Family smoking (ref. none)	Yes	1.82 *	1.16–2.88	1.62 *	1.06–2.50	1.22	0.77–1.92	0.81	0.51–1.29
**MOSCOW**		**Third tertile DP1**		**Third tertile DP2**		**Third tertile DP3**		**Third tertile PAP**	
		**OR**	**95% CI**	**OR**	**95% CI**	**OR**	**95% CI**	**OR**	**95% CI**
Sex, (ref. women)	Man	1.48	0.98–2.24	3.80 *	2.42–5.96	0.61 *	0.40–0.92	0.69	0.45–1.07
Возраст (реф. 10–12 лет)	13–15 лет	1.00	0.60–1.67	0.99	0.58–1.67	1.88 *	1.11–3.18	10.74 *	5.39–21.40
	16–17 лет	1.48	0.87–2.54	0.82	0.46–1.45	1.62	0.92–2.84	12.60 *	6.13–25.89
Living conditions, (ref. separate room)	Shared room	1.56	0.99–2.48	1.05	0.64–1.73	0.93	0.58–1.51	0.78	0.47–1.30
Family, (ref. Only one parent)	Complete	0.85	0.51–1.40	0.49 *	0.29–0.82	0.78	0.47–1.29	0.95	0.55–1.62
Number of children, (ref. One child)	Two or more	0.92	0.60–1.42	0.67	0.42–1.06	1.09	0.71–1.67	0.95	0.61–1.50
Mother's education (ref. Not higher)	Higher	0.71	0.43–1.17	0.64	0.38–1.08	1.38	0.82–2.34	0.78	0.45–1.35

Family smoking (ref. none)	Yes	1.81 *	1.18–2.76	2.42 *	1.54–3.82	0.88	0.57–1.35	0.50 *	0.32–0.79
**MURMANSK**		**Third tertile DP1**		**Third tertile DP2**		**Third tertile DP3**		**Third tertile PAP**	
		**OR**	**95% CI**	**OR**	**95% CI**	**OR**	**95% CI**	**OR**	**95% CI**
Sex, (ref. women)	Man	0.55 *	0.34–0.90	1.92 *	1.18–3.11	0.70	0.43–1.14	0.95	0.58–1.57
Возраст (реф. 10–12 лет)	13–15 лет	1.21	0.68–2.17	1.01	0.56–1.78	1.45	0.82–2.56	3.14 *	1.59–6.19
	16–17 лет	1.19	0.61–2.30	1.42	0.75–2.68	0.73	0.38–1.43	4.45 *	2.18–9.56
Living conditions, (ref. separate room)	Shared room	1.17	0.65–2.11	0.87	0.49–1.57	2.04 *	1.14–3.65	1.19	0.65–2.18
Family, (ref. Only one parent)	Complete	1.75	0.91–3.35	1.04	0.57–1.89	0.56	0.31–1.01	0.50 *	0.28–0.91
Number of children, (ref. One child)	Two or more	0.91	0.55–1.49	0.88	0.54–1.42	1.31	0.81–2.15	1.10	0.67–1.82
Mother’s education (ref. Not higher)	Higher	1.01	0.61–1.68	0.70	0.43–1.14	0.61 *	0.37–0.99	1.39	0.82–2.35
Family smoking (ref. none)	Yes	1.23	0.74–2.06	1.08	0.66–1.78	2.07 *	1.26–2.42	1.16	0.69–1.96

Note: in all models, adjustments were made for individual indicators: sex, age, city, living conditions, family size, number of children in the family, mother's education, family smoking; *—level of statistical significance ≤0.05
